# Microtubule-Associated Protein 4 Is a Prognostic Factor and Promotes Tumor Progression in Lung Adenocarcinoma

**DOI:** 10.1155/2018/8956072

**Published:** 2018-03-18

**Authors:** Xiaochun Xia, Chao He, Anqing Wu, Jundong Zhou, Jinchang Wu

**Affiliations:** ^1^Department of Radiation Oncology, Nanjing Medical University Affiliated Suzhou Hospital, Suzhou, Jiangsu 215001, China; ^2^Department of Radiation Oncology, Nantong Tumor Hospital, Affiliated Tumor Hospital of Nantong University, Nantong, Jiangsu 226361, China; ^3^Suzhou Cancer Center Core Laboratory, Nanjing Medical University Affiliated Suzhou Hospital, Suzhou, Jiangsu 215001, China; ^4^School of Radiation Medicine and Protection, Soochow University, Suzhou, Jiangsu 215123, China

## Abstract

Microtubule-associated protein 4 (MAP4) plays an important role in microtubule assembly and stabilization. The purpose of this study was to investigate the level of expression of MAP4 in lung adenocarcinoma (LADC) samples and to evaluate its prognostic value and the influence on cancer progression in LADC patients. The expression of MAP4 protein was analyzed using immunohistochemistry. The clinical significance and the prognostic significance of MAP4 expression were assessed by Kaplan-Meier analysis and Cox regression analysis. The roles of MAP4 in the migration and invasion of LADC cells were detected by wound-healing assays and transwell assays, respectively. We found the expression levels of MAP4 protein in LADC tissues to be significantly higher than those in noncancerous tissues. MAP4 expression was significantly correlated with differentiation, pathological T stage, and TNM stage. Kaplan-Meier survival analysis indicated that patients with high MAP4 expression had significantly poorer overall survival (OS). Cox regression analysis revealed that MAP4 expression level was an independent prognostic factor for OS. Functionally, in vitro studies showed that MAP4 knockdown efficiently suppressed the migration and invasion of LADC cells. Our data indicated that MAP4 protein may represent a novel prognostic biomarker and a potential therapeutic target for LADC.

## 1. Introduction

Cancer is an enormous public health burden worldwide. More than 14 million estimated new cancer cases and 8 million cancer deaths occurred in 2012. Lung cancer remains the leading cause of cancer mortality for humans all over the world [1], and non-small-cell lung carcinoma (NSCLC) is the predominant type of lung cancer, making up 85% of all cases [2]. Lung adenocarcinoma (LADC) is a type of NSCLC that has come to make up a growing proportion of NSCLC in recent years. Radical resection is the principal treatment for the patients with stage I–IIIa NSCLC, but the 5-year survival rate remains low. The high mortality rate is largely attributed to local recurrence and distant metastasis of NSCLC [3]. A number of randomized clinical trials demonstrated that adjuvant chemotherapy is the standard treatment for resected early-stage NSCLC patients [4–6], but few patients benefit from the treatment. Hence, identification of novel prognostic biomarkers relating to cancer recurrence and metastasis is critical to improving the treatment strategy for NSCLC patients.

Microtubule-associated proteins (MAPs) have many subtypes, including MAP1A, MAP1B, MAP2, MAP4, and tau proteins. MAP4 is mainly expressed in nonneuronal tissues and ubiquitously found in all cell types [7]. Heat-stable MAP4 is composed of an asymmetric structure with an N-terminal projection (PJ) domain and a C-terminal microtubule-binding (MTB) domain. Studies have shown that the PJ domain takes part in the regulation of the dynamic instability and the phosphorylation of MTB domain participates in the cell cycle progression [8–11]. MAP4 has been reported to play an important role in the modulation of microtubule dynamics through interaction with septin [12]. It has been reported that MAP4 has effects on paclitaxel resistance and the rapid progression of apoptosis by the negative regulation of p53 [13–17]. Until now, there are still few studies of the relationship between MAP4 and human cancers. Ou and colleagues have reported that the cAMP/PKA signaling pathway is involved with bladder cancer cell invasion by targeting MAP4-dependent microtubule dynamics [18]. Recently, overexpression of MAP4 has been demonstrated to be associated with poor prognosis and promotion of cell invasion and migration through MAP4-ERK-Jun-VEGF signaling in esophageal squamous cell carcinoma [19].

Up to now, however, no reports have been published on the relationship between MAP4 expression and clinicopathological features and prognosis of LADC patients. In this study, we demonstrated that expression of MAP4 was closely correlated with LADC progression and poor prognosis by promoting cancer cell invasion and migration.

## 2. Materials and Methods

### 2.1. Patients and Tissue Samples

Fresh human LADC tissues and adjacent tissues used for this study were obtained from surgical resection specimens collected by Nanjing Medical University Affiliated Suzhou Hospital (Jiangsu, China). None of the patients received any treatment before surgery. The tissue samples were immediately snap-frozen and stored at −80°C for histological examination after surgery. Clinicopathologic parameters (Table 1) and OS data were collected. 91 LADC patients were followed up until August 2014 or until death, with a median follow-up period of 39 months (range 1–121 months). All patients signed informed consent, and the study was approved by the Institutional Ethics Committee of Nanjing Medical University.

### 2.2. Immunohistochemistry

Paraffin-embedded LADC tissue samples and cancer adjacent tissues were cut into 4 *μ*m thick sections and affixed to the slides. The tissue sections were deparaffinized in xylene and rehydrated in a graded series of ethanol solutions using standard procedures. The sections were subsequently submerged in EDTA (pH 8) and autoclaved at 121°C for 5 min to retrieve the antigenicity. After washing in TBS, endogenous peroxidase was blocked by incubation in 3% hydrogen peroxide solution in methanol for 10 min at room temperature. Then, incubation with MAP4 antibody (Proteintech, Rosemont, IL, US) diluted at 1 : 4000 in TBS containing 0.5% BSA was carried out at 4°C overnight followed by further washing with buffer to remove unbound antibody. The sections were incubated with Envision secondary antibody (DAKO, Santa Clara, CA, US) for 30 min at room temperature. Chromogen (DAB) (GeneTex, Irvine, CA, US) was added to visualize the reaction. The sample was then counterstained with commercial hematoxylin (Beyotime Biotechnology, Nantong, Jiangsu, China), dehydrated sequentially in alcohols and xylene, and mounted.

### 2.3. Immunohistochemical Staining Evaluation

Two experimenters who were blinded to clinicopathologic information and patient outcomes evaluated the slides independently. MAP4 staining was mainly localized in the cytoplasm. MAP4 expression was quantified using a visual grading system based on the extent of staining and the intensity of staining. The percentage of positive-staining tumor cells was scored as follows: 0 (negative), 1 (1–30%), 2 (31–60%), and 3 (>60%). Staining intensity was scored as follows: 0 (none), 1 (weak staining), 2 (moderate staining), and 3 (strong staining) [20]. The final score was calculated by multiplying the extent of staining score by staining intensity score. Scores > 4 were considered indicative of high expression levels. Cases with discrepancies were rereviewed simultaneously, and consensus decisions were made.

### 2.4. Cell Culture

Human LADC cell lines A549 and H1299 were obtained from the Shanghai Cell Bank (Shanghai, China). A549 cells were cultured in DMEM medium, and H1299 cells were cultured in RPMI-1640 medium supplemented with 10% fetal bovine serum (FBS) (HyClone, Waltham, MA, US), penicillin (100 U/ml), and streptomycin (100 mg/ml) in a humidified atmosphere, with 5% CO_2_ at 37°C.

### 2.5. Reverse Transcription-Polymerase Chain Reaction (RT-PCR)

Total RNA was extracted using TRIzol reagent (Invitrogen, Carlsbad, CA, US) in accordance with the manufacturer's instructions. The concentrations of RNA were determined using a NanoDrop2000 (NanoDrop, US). For reverse transcription, 1.0 *μ*g of RNA/sample was reverse-transcribed using an oligo(dT)_12_ primer and SuperScript II reverse transcriptase (Invitrogen, US) according to the manufacturer's instructions. The primers for MAP4 and *β*-actin were as follows: forward, 5′-CCGGGAACTCAGAGTCAAAGA-3′ and reverse, 5′-CTCCATGACCACCATTGGCT-3′ (MAP4); forward, 5′-AGCGAGCATCCCCCAAAGTT-3′ and reverse, 5′-GGGCACGAAGGCTCATCATT-3′ (*β*-actin). RT-PCR analysis was performed with the StepOne Plus instrument (Applied Biosystems, Waltham, MA, US) using the 2X Taq PCR Master Mix (Takara, Dalian, Liaoning, China) according to the manufacturer's instructions. Human *β*-actin gene was used as an endogenous control. All samples were examined in triplicate. Relative mRNA levels were calculated based on the threshold cycle (Ct) values, and relative expression levels were calculated by using the 2-ΔCt method.

### 2.6. Western Blot Analysis

Cells were harvested and lysed in the radio immunoprecipitation assay (RIPA) lysis buffer that contained protease inhibitors for 20 min at 4°C. The proteins were separated by 10% SDS-PAGE and transferred to PVDF membranes (Millipore, Bedford, MA, US). After blocking with 5% nonfat milk in TBS-Tween-20 for 1 h at room temperature, the membranes were incubated with primary antibodies targeting *β*-actin (Beyotime Biotechnology, Nantong, Jiangsu, China) and MAP4 (Proteintech, Rosemont, IL, US). The membranes were incubated with a horseradish peroxidase- (HRP-) conjugated anti-rabbit or anti-mouse secondary antibody (Beyotime Biotechnology, Nantong, Jiangsu, China) for 2 h after washing three times with TBST. The proteins were visualized using the enhanced chemiluminescence (ECL) Western blot analysis system (Bio-Rad). *β*-actin protein expression was detected as the internal control.

### 2.7. Wound-Healing and Invasion Assays

Wound-healing assay was used to detect cell migration in vitro. Cells were seeded into 6-well plates. Five scrape wounds were drawn vertically with a pipette tip for each sample, when the cells were grown to confluence in 24 h. The floating and detached cells were washed three times with PBS before taking photos. The cells were photographed at 0, 24, and 48 h for A549, and 0, 24, and 72 h for H1299 using a light microscope (Leica Corporation, Wetzlar, Germany). 1 × 10^5^ cells (200 *μ*l) in serum-free medium were added to the upper chambers of the 24-well Transwell apparatus (Costar, New York, NY, US). The Transwell inserts were precoated with 40 *μ*l Matrigel (1 : 4 dilution; BD Biosciences, San Jose, CA, US). Medium containing 10% FBS was added to the lower chambers. The insert was washed with PBS, and the cells on the upper surface of the insert were removed by wiping with a cotton swab after incubation for 24 h at 37°C. Then, the inserts were fixed with 3.7% paraformaldehyde and stained with 2% crystal violet. At last, the invading cells in the lower chambers were photographed under a microscope and counted in three random fields at ×200 magnification.

### 2.8. Statistical Analysis

Values are presented as the means ± standard deviation (SD). The chi-square test or Fisher's exact test was carried out to evaluate the relationship between MAP4 expression and the clinicopathological variables. OS was calculated actuarially according to the Kaplan-Meier method and analyzed by the log-rank test. The univariate and multivariate Cox proportional hazards model was used to estimate hazard ratios and 95% confidence intervals for patient outcome. Statistical analysis was performed using the paired samples *t*-test and Student's *t*-test. All tests were 2-sided, and differences were considered significant when *P* < 0.05. Statistical analysis was performed using SPSS 16.0 (SPSS Inc., Chicago, IL, US).

## 3. Results

### 3.1. Association of MAP4 Protein Expression with Clinicopathological Features of LADC

The correlations between MAP4 expression and the clinicopathological features of LADC patients are shown in Table 1. The level of MAP4 expression was significantly closely correlated with differentiation (*P* = 0.03), pathological T stage (*P* < 0.01), and TNM stage (*P* < 0.01). However, there were no significant differences between MAP4 expression and other clinicopathological factors, including gender (*P* = 0.75), age (*P* = 0.10), and pathological N stage (*P* = 0.06).

### 3.2. MAP4 Expression in LADC Tissues and the Paired Adjacent Normal Lung Tissues

**Figure 1:**
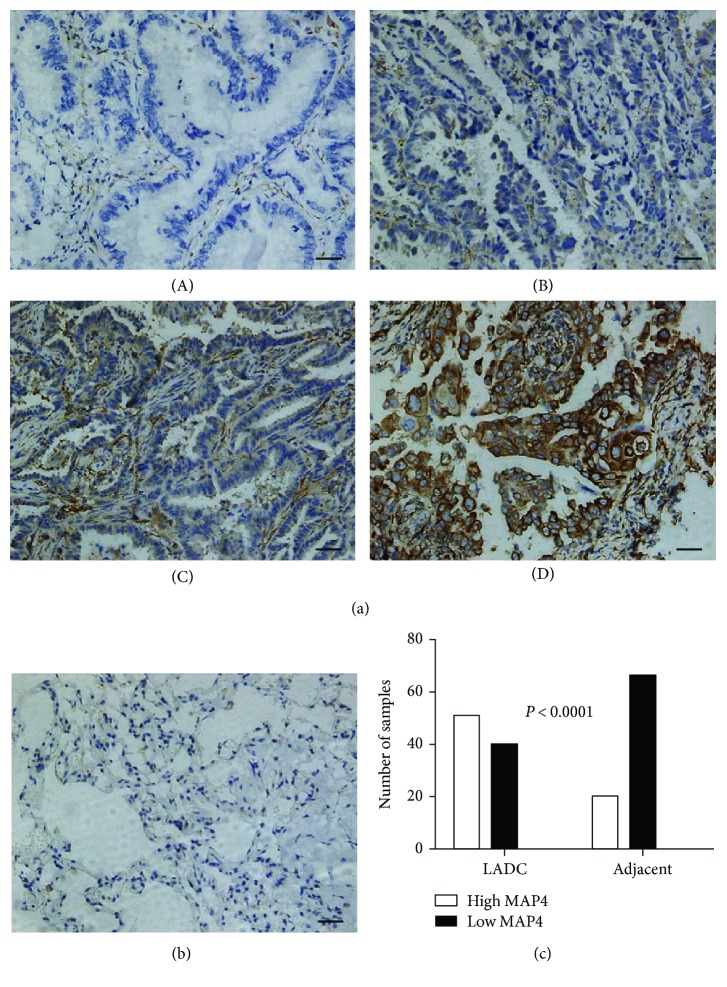
MAP4 expression in LADC tissues and adjacent normal lung tissues (bar = 100 *μ*m, 200x). (a) Immunohistochemical analysis of MAP4 in tumor tissue samples: (A) negative expression; (B) low expression; (C) moderate expression; (D) high expression. (b) Negative MAP4 expression in adjacent tissues. (c) MAP4 expression was examined by IHC in 91 LADC tissue samples and 86 matched adjacent normal lung tissue samples. MAP4 expression was significantly increased in tumor tissues compared with that in adjacent lung tissues (*P* < 0.0001, paired samples *t*-test).

The expression of MAP4 protein in paraffin-embedded, archived LADC tissue samples (*n* = 91) and the paired adjacent normal lung tissues (*n* = 86) were analyzed using immunohistochemistry. MAP4 staining in LADC tissues appeared as brown particles, which were mainly localized within the cytoplasm, accompanied by a stromal reaction (Figure 1(a)). Most of the normal carcinoma-adjacent tissues showed little or no staining (Figure 1(b)). The incidence of positive expression was 56.04% (51/91) in LADC tissues and 23.25% (20/86) in the normal tissues. The level of MAP4 expression was significantly higher in LADC than in normal lung tissues, as indicated by statistical analysis (*P* < 0.0001) (Figure 1(c)).

### 3.3. Survival Analysis Correlation of MAP4 Expression in LADC with Clinicopathological Characteristics

**Figure 2:**
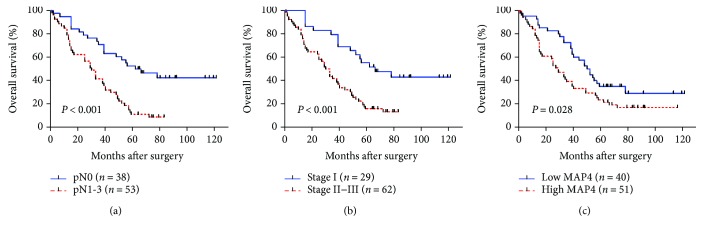
Clinicopathological parameter and MAP4 expression correlate with poor prognosis in human LADC patients. Kaplan-Meier overall survival curves for patients with N stage (a), TNM stage (b), and MAP4 expression levels (c) (log-rank test).

To investigate the prognostic value of MAP4 for LADC, we evaluated the relationship between MAP4 expression and OS in all patients with Kaplan-Meier analysis. The survival curve showed that patients with high levels of MAP4 expression had a significantly poorer OS than those with low levels of expression (log-rank test, *P* = 0.028) (Figure 2(c)), while lymphatic metastasis and higher TNM stage also had a significantly worse OS (log-rank test, both *P* < 0.001) (Figures 2(a) and 2(b)). Univariate and multivariate analyses were carried out to evaluate the impact of MAP4 expression and clinicopathological factors on the prognosis of LADC patients using Cox proportional hazard model. Pathological N stage (HR: 2.767; 95% CI: 1.697–4.512; *P* < 0.001), TNM stage (HR: 2.474; 95% CI: 1.517–4.036; *P* < 0.001), and MAP4 expression level (HR: 1.714; 95% CI: 1.060–2.771; *P* = 0.028) were significant prognostic factors of LADC in univariate analysis (Table 2). Subsequently, the factors with significant values in univariate Cox regression analysis were enrolled in multivariate analysis, which showed pathological N stage (HR: 2.770; 95% CI: 1.642–674; *P* < 0.001) and high expression of MAP4 (HR: 1.655; 95% CI: 1.017–2.692; *P* = 0.042) to be the independent prognostic factors for OS (Table 2).

### 3.4. MAP4 Promotes Cell Migration and Invasion in LADC Cells

**Figure 3:**
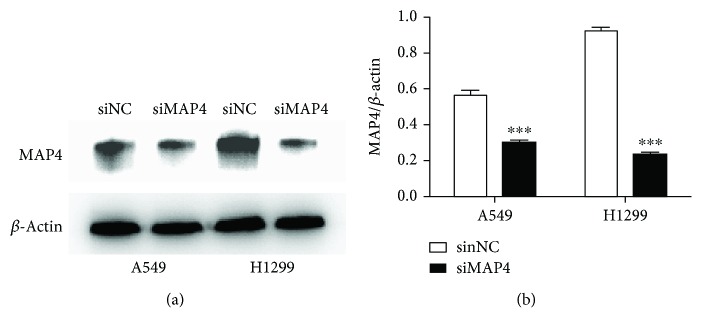
MAP4 expression was repressed by MAP4 siRNA in lung cancer cells. A549 and H1299 cells were transfected with siRNA against MAP4 and negative control sequence (siNC). Expression levels of MAP4 protein in siMAP4-transfected cells were significantly lower than those in siNC-transfected cells by Western blot analysis (a). Densitometry analysis of MAP4 expression in siMAP4-transfected A549 and H1299 cells (b). *β*-actin levels served as a loading control. The ratio of MAP4 expression to *β*-actin was calculated and represented as mean + SD relative to the control (^∗∗∗^*P* < 0.001, Student's *t*-test).

**Figure 4:**
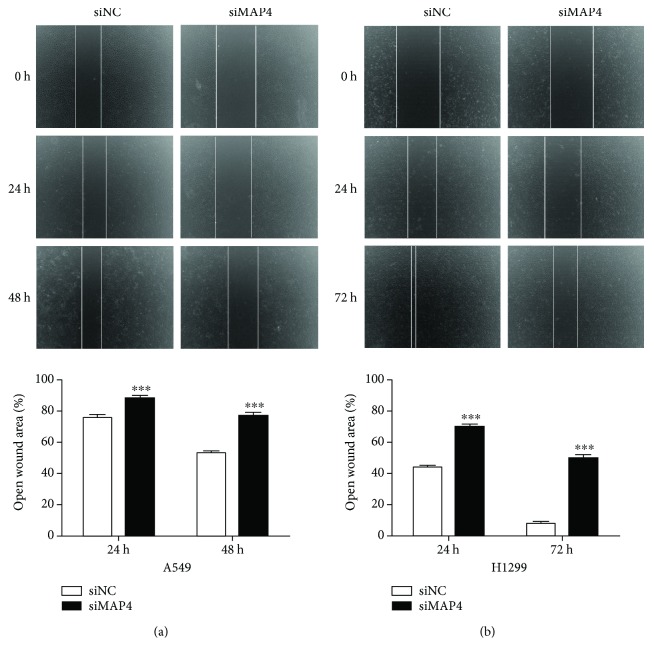
Effect of MAP4 knockdown on cell migration in vitro. Wound-healing assay was performed with both A549 (a) and H1299 (b) cells transfected with MAP4 siRNA or a negative control to examine the cell migration. The ability of cell migration was repressed after MAP4 knockdown in both A549 and H1299 cells. The open wound area was normalized to the area at the initial time (0 h). (a, b) Top: representative images. (a, b) Bottom: the percentage of filled wound area was calculated and represented as mean + SD relative to the control (^∗∗∗^*P* < 0.001, Student's *t*-test).

**Figure 5:**
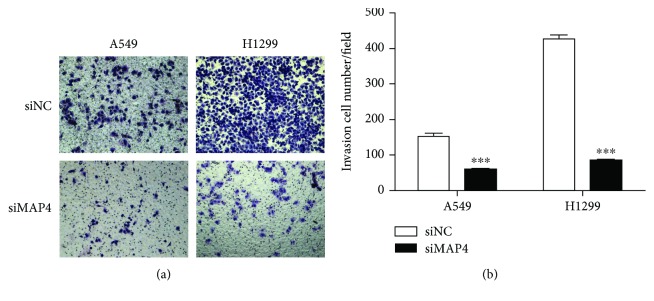
MAP4 promotes LADC cell invasion in vitro. The matrigel transwell assay was performed in A549 and H1299 cells transfected with MAP4 siRNA or a negative control to determine cell invasion potential. The number of cells migrated to the bottom were calculated manually. MAP4 knockdown repressed the cell invasion in both two types of cells. (a) Representative photos of invasive cells. (b) Quantification analysis of the invasive cell number per field. Data represent mean + SD of duplicates from three fields of view (^∗∗∗^*P* < 0.001, Student's *t*-test).

To determine the effects of MAP4 on cancer cell migration and invasion, we repressed MAP4 expression in A549 and H1299 cells using the specific MAP4-siRNA. The transfection efficiency was validated by Western blot analysis of cell lysates. The expression of MAP4 was inhibited effectively after transfected with MAP4-siRNA (Figure 3). Wound-healing assays and Boyden chamber transwell assays were performed to measure the response of two LADC cell lines to the MAP4-siRNA. The motility of cells was determined at different times in wound-healing assays (24 h and 48 h for A549 cells; 24 h and 72 h for H1299 cells). As shown in Figures 4(a) and 4(b), knockdown of MAP4 slowed down the migration of A549 and H1299 cells significantly as compared to the control cells (all *P* < 0.001). Meanwhile, the results of matrigel transwell assays indicated that the downregulation of MAP4 reduced the number of invasion cells in both cell lines below control cell levels (all *P* < 0.001) (Figure 5). These results suggested that MAP4 may be involved in the migration and invasion of LADC cells.

## 4. Discussion

Lung adenocarcinoma is the most common subtype of NSCLC, making up about 80% of all diagnosed lung cancers [21]. Local recurrence and distant metastasis are the principal causes of deaths in LADC patients [3]. Molecular targeted therapy has developed rapidly in the past decade. Compared with the traditional chemoradiotherapy, targeted therapy significantly improved the long-term survival rate of lung adenocarcinoma patients [22, 23]. However, about half of lung cancer patients are not suitable for targeted therapy because of the absence of known driver mutations [24]. A potential biomarker that can predict responses to therapy and inform individualized treatment strategies and novel insights into the mechanism underlying LADC pathogenesis is critically needed. In this study, we demonstrated that MAP4 is an independent prognostic factor of LADC with the ability to promote cancer cell migration and invasion.

MAP4, a type of microtubule-associated proteins, was first identified in mouse neuroblastoma cells [25]. Studies have reported that MAP4 plays an important role in microtubule assembly and stabilization [8, 11, 12]. In the process of cancer chemotherapy, an antimicrotubule drug is administered safely at a time, and cancer cells are more sensitive to DNA-damaging agent when MAP4 is downregulated [26]. Researchers have found that the ratio of *MAP4* to *stathmin* mRNA was higher in lung cancer tissues than in normal lung tissues, suggesting that the ratio might be a clinically relevant biomarker for NSCLCs [27]. In prostate cancer, MAP4 was also reported to be a potential biomarker for detection of prostate cancer and discrimination between prostate tumors with different malignancy and aggressiveness [28]. Downregulation of MAP4 is associated with poor differentiation and proliferation in primary oral squamous cell carcinomas [29]. The novel MALT1-MAP4 fusion protein, which is different from the known MALT1-associated chromosomal rearrangements, may be a new pathogenetic reason of diffuse large B-cell lymphoma [30]. However, few studies have focused on the relationship of MAP4 function with cancer progression or poor prognosis in human cancer.

In the present study, we hypothesized that aberrant MAP4 expression may be involved in the progression of LADC. LADC tissue samples and the adjacent morphological normal lung tissues were collected to assess alterations in MAP4 expression. We found MAP4 expression to be significantly higher in the LADC tissues than in the normal controls. This finding strongly suggested that elevated MAP4 expression may play an oncogenic role in promoting tumor progression. Subsequently, the clinical significance of MAP4 expression in 91 LADC patients was analyzed and the MAP4 high-expression frequency was 56.04%. According to clinicopathologic characteristics, high levels of MAP4 expression was significantly closely correlated with differentiation, pathological T stage, and TNM stage but not with gender, age, or pathological N stage. Moreover, Kaplan-Meier survival curves showed that the patients with lymphatic metastasis, higher TNM stage, and elevated MAP4 expression had a significantly poorer OS. Cox regression analysis revealed that pathological N stage and MAP4 expression were independent prognostic factors of OS in LADC patients. We also found that MAP4 knockdown reduced cell migration and invasion in vitro studies. Collectively, these results strongly demonstrated that elevated MAP4 expression is associated with the increased metastatic potential of cancer cell and worse long-term survival of LADC patients. Although the underlying mechanism is unclear, the evidence seems to indicate that MAP4 is an important regulator during cancer progression. Our new findings were consistent with recent studies, which have shown a correlation between MAP4 overexpression and poorer prognostic outcome in bladder cancer and esophageal squamous cell carcinoma [18, 19].

In summary, our present data demonstrated that the elevated expression of MAP4 was associated with differentiation, pathological T stage, and TNM stage in LADC tissues. Hence, MAP4 may be an independent prognostic factor for poor prognosis of LADC. The present study has some limitations, such as the small sample size and the unknown potential molecular mechanism between aberrant MAP4 expression and cancer progression in LADC. These findings remain to be confirmed by more samples and further studies. Based on these data, we propose that MAP4 may have the role of identifying the subgroup of patients with more aggressive tumors and poor prognostic outcome and it may become an attractive and promising therapeutic target for LADC patients in the future.

## Figures and Tables

**Table 1:** Correlation between MAP4 protein expression level and clinicopathological variables of patients with lung adenocarcinoma.

Variables	*N*	MAP4 expression (%)		*χ* ^2^	*P* value
		High	Low		
Gender				0.10	0.75
Male	49	30 (61.22)	19 (38.78)		
Female	42	21 (50.00)	21 (50.00)		
Age (year)				2.69	0.10
≤60	40	23 (57.50)	17 (42.50)		
>60	51	28 (54.90)	23 (45.10)		
Differentiation				4.45	0.03
Well/moderate	65	36 (55.38)	29 (44.62)		
Poor	26	15 (57.69)	11 (42.31)		
pT				8.10	<0.01
T1-2	69	40 (57.97)	29 (42.03)		
T3-4	22	11 (50.00)	11 (50.00)		
pN				3.45	0.06
N0	38	20 (52.63)	18 (47.37)		
N1-3	53	31 (58.49)	22 (41.51)		
TNM stage				9.31	<0.01
I	29	14 (48.28)	15 (51.72)		
II-III	62	37 (59.68)	25 (40.32)		

*N*: number; MAP4: microtubule-associated protein 4; p: pathological staging; TNM: tumor-node-metastasis.

**Table 2:** Univariate and multivariate Cox regression analysis for OS in lung adenocarcinoma patients.

Variates	Categories	Univariate analysis		Multivariate analysis	
		HR (95%CI)	*P* value	HR (95%CI)	*P* value
Gender	Male versus female	1.361 (0.843–2.199)	0.207		
Age	>60 versus ≤60 years	1.058 (0.654–1.711)	0.818		
Differentiation	Poor versus well/moderate	1.025 (0.602–1.746)	0.927		
pT	T3-4 versus T1-2	1.393 (0.771–2.516)	0.272		
pN	N1-3 versus N0	2.767 (1.697–4.512)	<0.001	2.770 (1.642–4.674)	<0.001
TNM stage	II-III versus I	2.474 (1.517–4.036)	<0.001		
MAP4 expression	High versus low	1.714 (1.060–2.771)	0.028	1.655 (1.017–2.692)	0.042

OS: overall survival; HR: hazard ratio; CI: confidence interval; p: pathological staging; TNM: tumor-node-metastasis; MAP4: microtubule-associated protein 4.
